# Research on the Roundness Approximation Search Algorithm of Si_3_N_4_ Ceramic Balls Based on Least Square and EMD Methods

**DOI:** 10.3390/ma16062351

**Published:** 2023-03-15

**Authors:** Jian Sun, Wei Chen, Jinmei Yao, Zhonghao Tian, Longfei Gao

**Affiliations:** 1 School of Mechanical Engineering, Shenyang Jianzhu University, Shenyang 110168, China; 2 National-Local Joint Engineering Laboratory of NC Machining Equipment and Technology of High-Grade Stone, Shenyang 110168, China

**Keywords:** silicon nitride ceramic ball, uniform envelope of the lapping trajectory, empirical mode decomposition method, least square method, fine processing, roundness deviation

## Abstract

This paper aims to obtain the best shape accuracy evaluation algorithm for silicon nitride ceramic balls after lapping, and to extract the initial signal of the ball surface to improve the accuracy and reliability of the algorithm. The research methods of this paper are as follows: Firstly, an analysis of the uniform envelope of the lapping trajectory of ceramic balls is carried out to verify whether the lapping trajectory after processing can achieve a consistent envelope on the balls’ surface. On this basis, it is found through experiments that the standard deviation SD between the roundness deviations of different contour sections is small. The value is maintained at approximately 0.03 μm, and the roundness deviation can approximately replace the spherical deviation. Then the different contour sections of the sphere are sampled by the Taylor roundness instrument. Considering the uncertainty, the sampling points of different contour sections are averaged and used as the original signal of the sphere surface. Then the EMD method is used to process the signal to be detected on the sphere surface. The initial signal of the sphere surface is extracted by judging whether the number of ripples *K*c obtained by decomposition is greater than the critical value. Then the initial signal is used as the input value of the approximation algorithm. Through the roundness deviation approximation algorithm based on the least square method, the given minimum approximation domain range is finely processed. The divided fine points are used as the center of the circle to intersect with the initial signal. The maximum, minimum, and range of each circle are calculated to obtain the roundness error based on the minimum circumscribed circle, the maximum inscribed circle, and the minimum region method. Finally, the calculated values are compared with those obtained by the traditional algorithm. The experimental results of this paper show that the algorithm is consistent with the roundness error measured by the instrument, compared with the mainstream evaluation criteria. In summary, the conclusions can be drawn as follows: Through a large number of experimental cases and comparative experiments, the algorithm has high accuracy and reliability. The research results of this paper have essential reference significance for accurately evaluating the shape accuracy of ceramic balls in actual production.

## 1. Introduction

With their superior performance, ceramic ball bearings occupy a position that cannot be ignored in many key technical fields. As a key component of the ceramic ball, its shape contour accuracy greatly affects the performance of bearings [1,2,3]. As an important basis for evaluating the rotation accuracy and interchangeability of bearings, higher spherical error and surface waviness deviation will lead to greatly reduced rotation accuracy and life of bearings. Therefore, how to improve the shape contour accuracy of ceramic balls and reduce their roundness deviation is one of the main technical difficulties and priorities in the current research on the accuracy of ceramic ball bearings [4,5,6,7,8].

**Figure 1:**
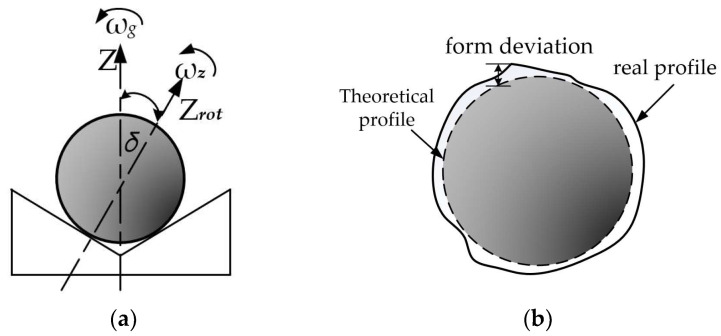
Ceramic ball lapping principle and spherical deviation schematic diagram. (**a**) Spherical Lapping Schematic; (**b**) Diagram of spherical deviation.

As shown in Figure 1, when the ceramic ball is lapping, the external device drives the lapping disc to rotate around the *Z* axis with the angular velocity *ω*_g_. While the lapping disc rotates, the ceramic ball in the track will produce an angular velocity ωz that rotates around its instantaneous rotation axis *Z*_rot_. The spatial angle between the angular velocity *ω*_g_ of the lapping disc and the angular velocity *ω*_z_ of the ceramic ball is set to *δ*. When the value of *δ* is constant, the lapping track on the surface of the ceramic ball fails to cover the whole ball evenly, and the motion track of the lapping disc on the surface of the ball is three concentric circles. At this time, the spherical deviation of the processed ball is large, and the roundness of the ball is poor. When the *δ* assignment is a variable, the lapping trajectory of the surface of the ceramic ball can be better enveloped in the whole ball [9,10]. At this time, the spherical deviation of the processed ball is smaller, and the roundness of the ball is better. The change of the spherical deviation of ceramic balls will also affect the change of material removal during processing. In theory, it is required to increase the material removal on the surface of ceramic balls when the spherical deviation is large and reduce the material removal on the surface of ceramic balls when the spherical deviation is small.

In order to make the shape contour of the ceramic ball after processing more accurately and with the surface spherical error smaller, many scholars at home and abroad have carried out in-depth and detailed research on it. On the basis of a three coordinate measuring machine, Wang Dongxia [11] improved the acquisition algorithm of spherical error contour data, which effectively reduced the spherical error in the process of measurement and evaluation. However, this study only compared the uncertainty of the MCM method and GUM method and did not point out the specific accuracy and error of each method in detail. Cai Zhen [12] proposed an improved algorithm based on the Cuckoo search algorithm, with high convergence speed and accuracy. The algorithm is currently effective in two-dimensional entities, but lacks experimental data in three-dimensional entities (ceramic balls). Zhang Ke and Wu Yu-hou [13,14] creatively put forward the conical groove lapping method, by improving the traditional V-groove lapping method. The experimental results show that the spherical error of ceramic balls processed by this method is the best when the rotation angle is controlled between 45° and 75°, which basically reaches the G5-level accuracy. Zhou Zhao-zhong et al. [15] proposed a spherical error correction model based on the spherical error of ceramic balls. Zhang [16] established a kinematic model based on the traditional lapping method, and analyzed the influence of the rotation angle, angular velocity, and angular velocity of the ceramic sphere on the shape of the sphere after processing. It is concluded that changing the above parameters during processing can form a more uniform lapping trajectory on the surface of the sphere. This conclusion provides a good theoretical basis for subsequent research. Zhang et al. [17] adjusted the axis offset of the upper and lower lapping discs in the traditional lapping method, to reduce the spherical error and improve the shape contour accuracy. Although this method can improve the spherical error of the sphere to a certain extent, the material removal method is mainly two-body removal, which has a great influence on the surface quality of the sphere. Lee RT [18,19] found that reducing the rotation angle of the sphere during the lapping process can effectively improve the spherical error by a large number of lapping experiments on the sphere. Meijian [20] proposed a progressive search method based on the minimum zone sphere (MZS), to evaluate the sphericity error. Although this method has high accuracy, the model is cumbersome, and there are many constraints in its practical application. Shi et al. [21] proposed a whale optimization algorithm to solve the sphericity calculation model. Although the calculation method of spherical error has certain advantages, the results easily fall into the local extremum in the iterative process, which affects the calculation accuracy. Damian Gogolewski [22,23] and his team creatively proposed to evaluate the contour by combining the Fourier transform and wavelet transform. This method has high accuracy and reliability, but there is a lack of application examples for ceramic balls. Therefore, Ito et al. [24] measured the spherical error of the probe tip of the coordinate measuring machine (CMM) by rotating the reference ball. The results show that the measurement uncertainty of this method is less than 0.5 μm; Jiang et al. [25] proposed an improved method to evaluate the spherical error based on the traditional bee colony algorithm. The convergence accuracy of this method is high, but it is easy to be limited to the optimal local solution.

Huang Fugui, Lei Xianqing et al. [26,27] innovatively proposed the region search method and the grid search algorithm, based on the traditional roundness algorithm least squares method. This kind of algorithm can avoid the complexity of solving nonlinear equations to a large extent and the inaccuracy of sampling points in special cases. However, this method is only applicable to the roundness deviation calculation model under the least square method; Yue et al. [28] proposed a roundness deviation calculation method suitable for the minimum inclusive region model. This method is based on geometric optimization. Although it has a wide range of applications, it is only suitable for small batch roundness deviation measurement because of its complex calculation.

As far as the current research status is concerned, there are many areas for improvement in the roundness evaluation of ceramic balls by mainstream research methods. This is mainly reflected in the following aspects [29]:

(1) In analyzing the spherical errors of ceramics, there is a lack of effective extraction of the detection signal, which leads to the interference of other signals except for the spherical error signal;
(2) The evaluation method of the spherical error of the ceramic ball lacks the corresponding model support, and most of the existing models focus on the accuracy optimization of the sampling points. There are few studies on the ball after lapping;
(3) At present, there is no uniform international standard for the definition of sphericity error, and the measurement of its accuracy is usually reflected by roundness error.

Because of the problems existing in the current research, this paper takes a silicon nitride ceramic ball as the research object. On the basis of studying the envelope of its lapping trajectory, a new spherical deviation calculation method suitable for the ceramic ball is proposed by combining the roundness approximation algorithm based on the least square method with the EMD method.

## 2. Analysis of Trajectory Uniform Envelope on the Surface of a Ceramic Sphere

Whether the ceramic ball can form a finished product with slight spherical deviation in the processing process depends on whether the lapping trajectory generated during rotation can evenly envelop the ball’s surface. Therefore, this paper includes the lapping trajectory of the sphere surface into the research scope and focuses on analyzing its influence mechanism [30,31].

**Figure 2:**
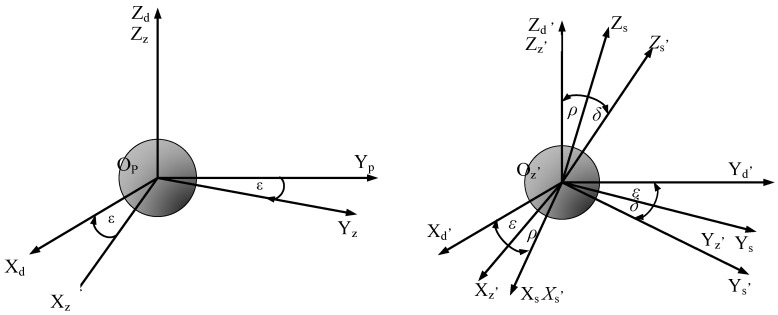
Transformation of coordinate system of ceramic ball motion.

To further study the motion state of the ceramic ball lapping into a ball, the fixed coordinate system and the revolution coordinate system of the ceramic ball in the initial state is considered to be converted into a local coordinate system with the center of the ball as the coordinate origin. The initial motion coordinate system and the transformed local coordinate system are shown in Figure 2.

From the analysis of Figure 2, it can be seen that the fixed coordinate system [*O*p *X*d *Y*d *Z*d] rotates ε degree with the revolution axis as the reference to obtain the revolution coordinate system [*O*p *X*z *Y*z *Z*z]. After the above two coordinate systems are transformed, the new coordinate systems are obtained as [*O*z, *X*d, *Y*d, *Z*d,] and [*O*z, *X*z, *Y*z, *Z*z,], respectively. The new revolution coordinate system [*O*z, *X*z, *Y*z, *Z*z,] is obtained by rotating ρ angle with Yz as the reference, and the rotation coordinate system of the ceramic ball is [*O*z, *X*s, *Y*s, *Z*s].

Setting the rotation angle of the ceramic ball as *δ*, the rotation coordinate system [*O*z, *X*s, *Y*s, *Z*s] is rotated by *δ*° around the rotation axis *Z*s of the ball, and the corresponding coordinate system under the change of the rotation angle is [*O*z, *X*s, *Y*s, *Z*s].

For the lapping trajectory of the ceramic ball surface, the discrete method is used to process it, and a model with high precision and good stability can be obtained. Specifically, the method is to uniformly sample the lapping trajectory of the outer surface of the ceramic ball after lapping. After determining the initial position, sampling interval, and sampling time, all the calculated sampling coordinates are mapped to the ball’s surface to obtain the corresponding lapping trajectory.

As an important prerequisite for the operation of the above discretization method, the initial position of the ceramic ball is usually determined by the original coordinates of the three contact points of the ceramic ball, the flat lapping disc, and the groove lapping disc. For the purpose of simplifying the subsequent calculation, this paper considers merging the above initial point coordinate model into a new matrix; the initial matrix *P*_N_ is as follows:

(1) $$P_{N}=\left(\begin{array}{ccc} r_{a}\cos\theta & r_{a} & 0\\ 0 & 0 & 0\\ r_{a}\sin\theta & 0 & r_{a} \end{array}\right)$$

The initial matrix *P*_N_ is transformed into a local coordinate system centered on the rotation axis of the sphere, and the matrix *P*_N_ is obtained by rotating the *ρ* angle, as shown below:

(2) $${P_{N}}^{\prime }=P_{N}\mathrm{tran}(u,\delta )$$

Among them, tran (*u*, *δ*) is defined as the coordinate change matrix of the ceramic ball, and its mathematical model is as follows:

(3) $$\mathrm{tran}(u,\delta )=\left(\begin{array}{ccc} u_{x}u_{x}(1-\cos(\delta ))+\cos\delta & u_{y}u_{x}(1-\cos(\delta )) & u_{x}u_{z}(1-\cos(\delta ))+\sin\rho \sin\delta \\ u_{x}u_{y}(1-\cos(\delta )) & u_{y}u_{y}(1-\cos(\delta ))+\cos\delta & u_{z}u_{y}(1-\cos(\delta ))-\cos\rho \sin\delta \\ u_{x}u_{z}(1-\cos(\delta ))-\sin\rho \sin\delta & u_{y}u_{x}(1-\cos(\delta )) & u_{z}u_{z}(1-\cos(\delta ))+\cos\delta \end{array}\right)$$

In this model, *u* is a related determinant of the change angle *ρ*, and its related expression is as follows:

(4) u=[cosρ sinρ 0 ]

On the basis of the initial position matrix *P*_N_, it is necessary to determine the angular velocity of the above three contact points, respectively. According to the relevant literature [32], the angular velocities *ω*_1_, *ω*_2_, and *ω*_3_ of the above points satisfy the following relationship:

(5) $$\left\{ \begin{array}{l} \omega_{1}{=\omega }_{b}cos\delta \\ \omega_{2}{=\omega }_{b}sin\delta \\ \omega_{3}=\omega c \end{array}\right.$$

In the above formula, *ω*_b_ is the projection of the angular velocity of the ceramic ball *ω*_z_ on the *x*-axis, *ω*_c_ is the projection of the angular velocity of the ceramic ball on the *y*-axis, and *θ* is the initial phase angle of the ball.

In general, the relationship between the angular velocity of the ceramic ball ω_z_ and the angular velocity of the lapping device *ω* is as follows:

(6) $$\omega_{z}=\frac{(R_{B}+R_{C})\omega }{2r(1+\sin\eta )\cos\delta }$$

In the above formula, *R*_B_ and *R*_C_ are the distance from the contact point to the rotation radius of the ball, *r* is the radius of the ceramic ball, and *η* is the groove angle of the groove. From the angular velocity of the above three contact points in the local coordinate axis, the angle value of each coordinate axis rotation in the unit sampling time can be derived.

(7) $$\left\{ \begin{array}{l} \alpha_{x}\left(i\right)=\omega_{z}(i)\cos\delta (i)\Delta t\\ \alpha_{y}\left(i\right)=\omega_{z}(i)\sin\delta (i)\Delta t\\ \alpha_{z}\left(i\right)=-r\omega z(i)\omega u(i)\sin\left(\frac{\pi }{2}-\delta (i)+\theta \right)\Delta t \end{array}\right.$$

In the above formula, *α*_x_ (*i*), *α*_y_ (*i*), and *α*_z_ (*i*) are defined as the angle values of the sphere in the unit sampling time, that is, the rotation amplitude value, which can be expressed as matrix Δ*F* = (*α*_x_ (*i*), *α*_y_ (*i*), *α*_z_ (*i*)). The number *i* is the number of trajectory points obtained by sampling; *δ*_t_ is the unit sampling time of the trajectory points on the surface of the sphere.

When the angle of rotation in the unit time is known, the rotation angle of any sampling point can be deduced as follows:

(8) $$\left\{ \begin{array}{l} C_{x}\left(i+1\right)=\alpha_{x}\left(i\right)C_{x}\left(i+1\right)\\ C_{y}\left(i+1\right)=\alpha_{y}\left(i\right)C_{y}\left(i+1\right)\\ C_{z}\left(i+1\right)=\alpha_{z}\left(i\right)C_{z}\left(i+1\right) \end{array}\right.$$

Among them, *C*_x_ (*i* + 1), *C*_y_ (*i* + 1), and *C*_z_ (*i* + 1) are defined as the angles of the sampling points relative to the three reference coordinates. The corresponding matrix *C* can be expressed as the real-time state of the rotation angle of the sphere in the lapping process, which is expressed as the following formula:

(9) $$C=\left(\begin{array}{ccc} C_{x}\left(i\right) & C_{y}\left(i\right) & C_{z}\left(i\right) \end{array}\right)$$

According to the definition of the above matrix PN and the change of the rotation angle of any sampling point, the relevant model of the trajectory coordinate P_N_ (*i* + 1) obtained by turning the ceramic ball at any angle during the lapping process is as follows:

(10) $$P_{N\;}{\left(i+1\right)}^{\prime }=P_{N}(i+1)\left(\begin{array}{ccc} u_{x}u_{x}(1-\cos(c))+\cos c & u_{y}u_{x}(1-\cos(c)) & u_{x}u_{z}(1-\cos(c))+\sin\rho \sin c\\ u_{x}u_{y}(1-\cos(c)) & u_{y}u_{y}(1-\cos(c))+\cos c & u_{z}u_{y}(1-\cos(c))-\cos\rho \sin c\\ u_{x}u_{z}(1-\cos(c))-\sin\rho \sin c & u_{y}u_{x}(1-\cos(c)) & u_{z}u_{z}(1-\cos(c))+\cos c \end{array}\right)$$

In summary, the lapping trajectory of the surface of the ceramic ball is mainly affected by the rotation amplitude matrix Δ*F* during the lapping process of the ceramic ball. It can be found that the main influence parameter of the matrix is the rotation angle *δ* (*i*) of the ceramic ball at any point. In order to facilitate the value and operation of the subsequent simulation, *Δδ* is expressed by the variation of the rotation angle in unit time.

### Ceramic Ball Trajectory Envelope Simulation Analysis

**Figure 3:**
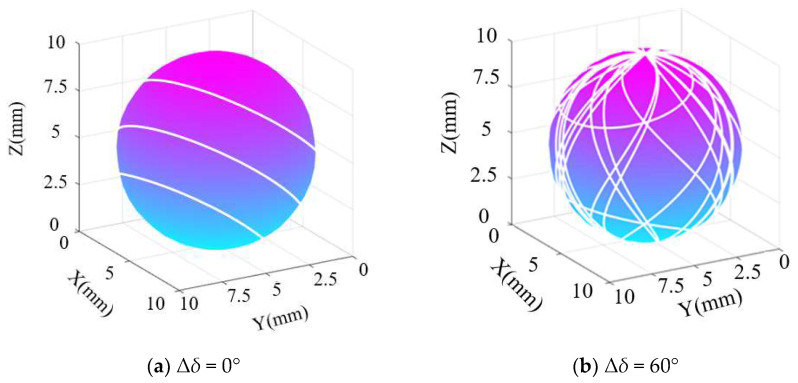

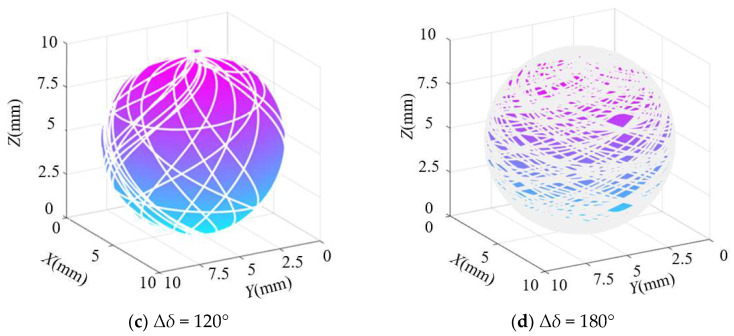
Lapping trajectory of sphere surface under different rotation angle variation.

From the previous establishment of the lapping trajectory model of the ball, it can be seen that the main influencing factor of the lapping trajectory of the ceramic ball surface is the rotation angle *δ*(*i*) of any point of the ball. Based on this, the simulation of this paper takes the silicon nitride ceramic ball as the research object and sets the initial blank ball diameter as D = 10 mm; the rotation speed of the lapping disc ng is 200 r/min, and the variation range of the rotation angle of the ceramic ball ∆*δ*(i) is set to four groups of 0°, 60°, 120°, and 180°. The simulation time is 1000 s, and the sampling step size is 0.001. Using MATLAB to simulate the lapping trajectory formed during the lapping of silicon nitride ceramic balls, the corresponding simulation results are shown in Figure 3.

It can be seen from Figure 3 that the lapping trajectory formed on the surface of the ceramic ball is three parallel lines when the change range of the rotation angle ∆*δ* = 0°, which is due to the contact point between the ball and the lapping disc during the lapping process. When the variation of the rotation angle is increased to 60°, the lapping trajectories begin to become dense, and multiple lapping trajectories are intertwined. When the rotation angle is further increased to 120°, the trajectory becomes denser. When the variation range of the rotation angle reaches 180°, the density of the trajectory reaches the maximum. At this time, the lapping trajectory of the sphere surface can basically envelop its surface evenly, which means that each section of the sphere surface can achieve more effective processing. Based on the simulation results, it can be seen that when the variation amplitude of the rotation angle of the ceramic sphere during the lapping process is as large as possible and the rotation angle is in a fully charged state, the surface of the sphere obtained by lapping can basically form a more uniform lapping trajectory, that is, the shape accuracy under different sections is basically consistent. This conclusion provides a theoretical basis for the subsequent model establishment of ceramic ball roundness error.

## 3. Basic Principle of Empirical Mode Decomposition

Empirical Mode Decomposition (EMD) is the main method to solve the difficulty of nonlinear and non-stationary signal extraction. The core of it is to decompose the detected signal layer by layer to the intrinsic mode function (IMF) with different characteristic vibration forms, and to screen out the signals that do not meet the functional conditions to avoid the influence of the stacking of signal waveforms on the decomposition results. Of course, the use of this method also has the corresponding theoretical premise [33]: (1) the Intrinsic Mode Function (the following abbreviation is IMF) is symmetrical about the time axis in the local range; and (2) the difference between the number of zeros and the number of poles of the IMF is no more than one.

Usually for time series signals, the decomposition steps of the EMD method are as follows [34,35,36]:

1. The collected signals are processed locally to obtain the maximum and minimum values in a fixed range. The maximum and minimum values obtained above are connected into upper and lower envelopes by cubic spline interpolation curve;
2. The data points corresponding to the upper and lower envelope lines obtained above are assigned, where the upper envelope line is set to *m*_1_, and the lower envelope line is set to *m*_2_; the mean value is then known (*m*_1_ + *m*_2_)/2. It can be seen from the relevant literature that the difference *D* between the time series signal and the mean value is an IMF, and the following formula can be obtained [37,38,39,40]:

(11) $$D=x(t)-\frac{m_{1}+m_{2}}{2}$$

For the difference *D*, it can usually be expressed as the initial Intrinsic Mode Functions obtained by decomposing the time series signal.

However, when decomposing the signals generated in the actual processing process, only obtaining a set of IMFs will affect the accuracy of the results. This is due to the phenomenon of underfitting and overfitting when interpolating the envelope curve through the cubic spline curve, resulting in multiple extreme points of the signal in the local atmosphere. Therefore, when processing the signal, the number k of the decomposition of the initial IMF should be increased as much as possible. After *k* times of decomposition, the corresponding difference *D*_K_ is expressed as the following model:

(12) $$D_{k-1}-m_{K}=D_{K}$$

Among them, *D*_K−1_ is the difference obtained by the *k* − 1 decomposition, and *m*_k_ is the mean value of the envelope obtained by the *k* decomposition.

3. It is judged whether the difference *D*_K_ obtained by *K*-times decomposition satisfies the theoretical conditions of the empirical mode decomposition method mentioned above. If it is not satisfied, the decomposition is continued until the difference *D*_K_ satisfies the condition that the IMF is established. The difference *D*_K_ obtained at this time is the initial IMF of the first group of the signal satisfying the condition, and its frequency is usually higher than other functions in the signal, denoted as *P*_1_(t):

(13) $$P_{1}\left(t\right)=D_{K}$$

4. Based on the obtained high-frequency Intrinsic Mode Functions *P*_1_(t), the corresponding low-frequency Intrinsic Mode Functions *Q*_1_(t) model can be derived as follows:

(14) $$Q_{1}(t)=x(t)-P_{1}\left(t\right)$$

5. The *m* − 1 decomposition of *Q*_1_(t) is performed by steps one to five, and the mth group of Intrinsic Mode Functions *P*_m_(t) and *Q*_m_(t) satisfying the conditions are obtained;
6. After obtaining all IMFs that meet the conditions, the decomposition process of the signal needs to be suspended. The relevant suspension function is as follows:

(15) $$L={\frac{\sum_{0}^{t}\left|D_{K-1}(t)-D_{K-1}(t)\right|}{\sum_{0}^{t}{\left|D_{K-1}(t)\right|}^{2}}}^{2}$$

In the formula, the value of L is the execution standard of the termination condition, and the critical value is limited to 0.2~0.3. When the signal is decomposed and when the value is less than the critical value, the process is terminated. The signal decomposition results are as follows:

(16) $$x(t)=\sum_{j}^{m}P_{j}\left(t\right)+Q_{m}\left(t\right)$$

In this formula, *P*_j_(t) is the data set from the initial high-frequency IMF to the minimum low-frequency IMF after multiple decompositions of the signal, that is, the IMF classification. The residual error after signal decomposition is *Q*_m_(t), which is usually expressed as the overall trend of the signal.

## 4. Ceramic Ball Roundness Deviation Extraction Based on EMD

### 4.1. Analysis of the Signal Composition of the Outer Surface of the Ceramic Ball

**Figure 4:**
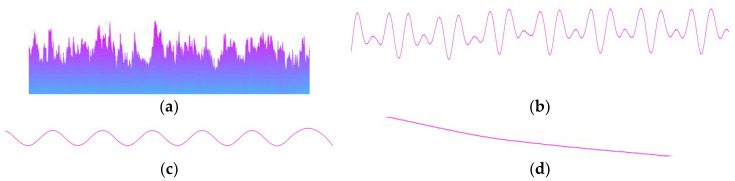
Signal to be measured on the outer surface of ceramic ball, including (**a**) signal to be measured, (**b**) surface roughness error signal, (**c**) waviness error signal, and (**d**) sphere form deviation signal.

The composition of the signal to be measured on the outer surface of the ceramic ball obtained by the actual lapping process is more complex. According to the current mainstream academic research point of view, it can be divided into the following two categories [41]:

1. Surface geometric accuracy error: As shown in Figure 4, the error is mainly caused by different lapping parameters set in the machining process. According to the collected signal frequency, it can be divided into high-frequency signal error, intermediate-frequency signal error, and low-frequency signal error. The high frequency-signal refers to the surface roughness error of the sphere, the intermediate-frequency signal refers to the waviness error of the outer surface of the sphere, and the low-frequency signal refers to the shape error of the sphere contour;
2. Measurement accuracy error: This error is mainly caused by external factors such as the accuracy of the measuring instrument, the accuracy of the measuring method, and the reliability of the measuring environment. It is usually manifested as a high-frequency signal interference error during the sampling process.

The roundness deviation of the ceramic ball is the main form of contour shape error, which is different from roughness error and waviness error. The signal frequency is mainly low-frequency, which is difficult to achieve by general signal extraction methods. The empirical mode analysis method can better reflect the frequency characteristics of the signal. Based on this, this paper uses the empirical mode analysis method to extract the roundness deviation signal of the ceramic ball.

### 4.2. Ceramic Ball Roundness Deviation Extraction Process

It can be seen from the previous article that the extraction of the roundness deviation of ceramic balls is based on the empirical mode analysis method. The specific process is as follows:

1. The collected signals of the outer surface of the ceramic ball are sorted and output as the original sequence *T*(t). The empirical mode decomposition method is used to decompose it into a series of IMF state functions with high-frequency, intermediate-frequency, and low-frequency characteristics. The low-order IMF state function in the decomposition result is the high-frequency part of the original signal, such as the surface roughness signal error of the ceramic ball and the measurement accuracy error, etc. The Intrinsic Mode Function between the low order and the high order is the intermediate-frequency part of the original signal, such as the surface waviness error signal of the ceramic ball. The remaining high-order Intrinsic Mode Function is the high-frequency part of the original signal, which is mainly the form deviation signal of the ceramic ball;
2. The number of waveforms of the decomposed signals is used as the main criterion to calculate the number of waveforms *K*_c_ of each Intrinsic Mode Function. Usually, the number of cycles *T* can be determined by calculating the zero-frequency component *Z*_j_(t) of the signal and the number of intersections n of the IMF, specifically, the number of waveforms *K*_c_ *= n/*2. For any Intrinsic Mode Functions, its zero-frequency component *Z*_j_(t) is expressed as follows:

(17) $$Z_{j}\left(t\right)=\sum_{j=1}^{m}P_{j}\left(t\right)$$

where *P*_j_(t) is the data set of the high-frequency to low-frequency IMF after the signal decomposition mentioned above, m is the number of signal sampling points in the decomposition process, and the number of intersections between the zero-frequency component and the IMF is usually an integer.

Compared with the target contour shape error signal, the waviness error signal and the roughness error signal belong to the middle- and high-frequency part in the outer surface detection signal of the ball, and the number of corresponding waveforms *K*_c_ is far greater than the number of roundness deviation signals. According to the relevant literature [41], the waviness error signal of the material surface after lapping usually has three manifestations; the number of waveforms *K*_c_ is 5, 15, and 45, respectively. In order to improve the accuracy of roundness deviation signal extraction, the waviness error signal with *K*_c_ value of 15 is set as the critical signal of accuracy error interference. The IMF with *K*_c_ greater than or equal to this value is the interference error that should be stripped. The remaining signal wave with *K*_c_ less than this value is the IMF containing the target form deviation signal.

3. The form deviation signal of the ceramic ball is obtained by reintegrating the extracted IMF and the residual signal error *Q*_m_ (t). The roundness deviation of the ceramic ball can be solved by applying the formula.

**Figure 5:**
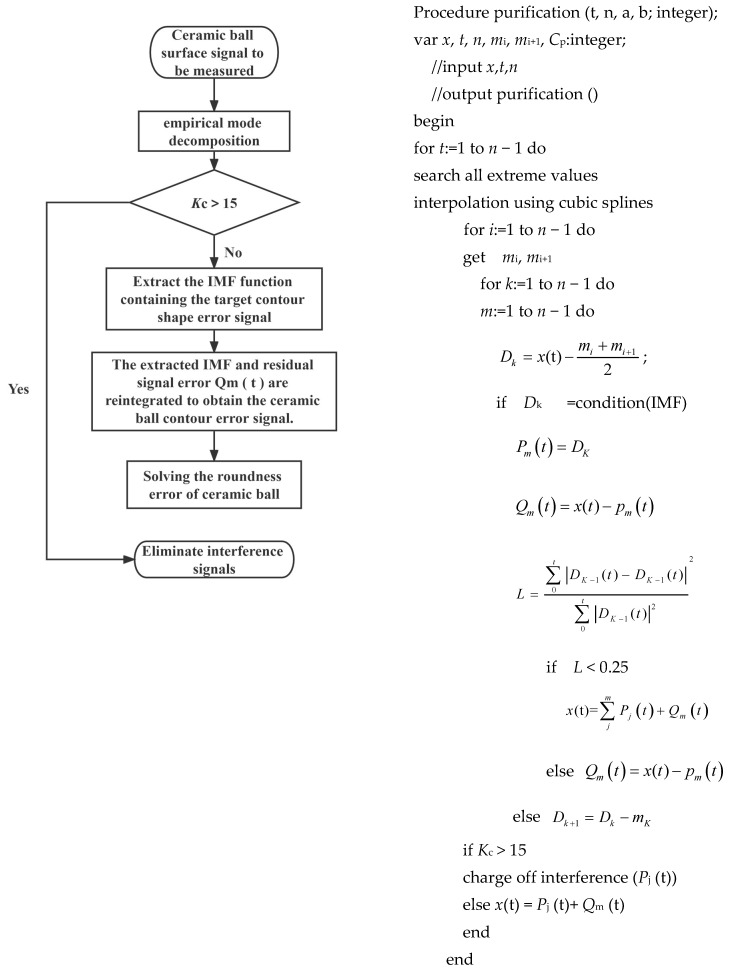
Ceramic ball surface roundness deviation signal extraction method.

The specific steps and pseudo-code of the above ceramic ball roundness deviation extraction method are as follows (Figure 5); the programming language used is MATLAB:

## 5. Ceramic Ball Roundness Approximation Algorithm

### 5.1. Roundness Deviation Model under Least Square Method

Under normal circumstances, there is a specific error between the outer contour circle and the ideal circle of the ceramic ball obtained by lapping, and it can be seen from the previous section that the shape accuracy of different sections of the ceramic ball obtained by the full change of the rotation angle is close to the same. Based on this, after using the EMD method mentioned above to extract the roundness error signal of the ball, it is proposed to use the contour section method for analysis. Specifically, the processed ball is cut along the axis, and the actual circle and the theoretical circle under the same cross-section are observed. The minimum distance between the two concentric circles is the roundness deviation [42].

At present, the calculation methods for the sphere mainly include (1) minimum zone method, (2) least square method, (3) minimum circumcircle method, and (4) maximum inscribed circle method [43]. In order to avoid the complexity of the model and the low accuracy of the predicted results, this paper uses the least square method to analyze the sphere.

The least square circle involved in this paper, as one of the current mainstream spherical evaluation methods, is widely used in precision measurement equipment with high reliability, good accuracy, and fast convergence [44]. In essence, the least square circle is used as the evaluation standard to calculate the sum of squares of the minimum distance between each position point on the measured outer contour and the least square circle. The premise of this method is to control the least square circle, that is, the circle as an ideal circle should conform to the minimum principle [45,46].

**Figure 6:**
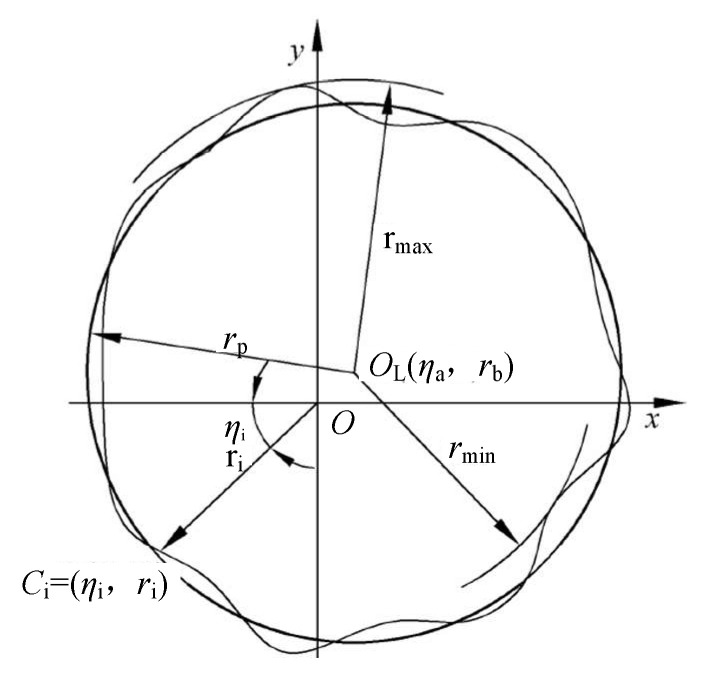
Diagram of least squares circle.

It can be seen from Figure 6 that the point *O*_L_ is the center of the least squares circle, and its coordinate is (*η*_a_, *r*_a_); ci is the actual measured contour point, and its corresponding coordinate in polar coordinates is (*η*_i_, *r*_i_); *r*_max_ and *r*_min_ are the radius values of the circle passing through the maximum and minimum points to be measured with the point *O*_L_ as the center. The center coordinates of the above polar coordinates are transformed into the corresponding values in the rectangular coordinate system, and the coordinates of OL are (*x*_a_, *y*_b_), where a and b are the center coordinates of the ideal contour; a and b satisfy the following relations, respectively:

(18) $$\left\{ \begin{array}{l} x_{a}=\frac{2}{n}\sum_{i=1}^{n}r_{i}\cos\eta_{i}\\ y_{b}=\frac{2}{n}\sum_{i=1}^{n}r_{i}\sin\eta_{i} \end{array}\right.$$

The specific mathematical model of this method is as follows:

(19) $$\left\{ \begin{array}{l} \Delta E\min=\mathrm{Min}\left|r_{\max}-r_{\min}\right|\\ r_{p}=e\cos(\eta_{i}-\beta )+\sqrt{\Delta r_{p}{}^{2}-{(\mathrm{esin}(\eta -\beta ))}^{2}} \end{array}\right.$$

The meanings of each symbol in the above formula are as follows: Δ*E*_min_ is defined as the roundness error of the sphere; e is the distance from the center of rotation *O*(x,y) to the center of least squares *O*_L_(*x*_a_,*y*_b_); *r*_p_ is the distance from any point on the actual contour line to the center of the sphere *O*_L_; *β* is the initial angle between *r*_p_ and the polar axis; and Δ*r*_p_ represents the distance deviation value in the model, which is defined as follows:

(20) $$\Delta r_{p}=r_{p}-(a\cos\eta_{i}+b\sin\eta_{i})$$

By integrating the above formulas, the mathematical model of the least squares roundness error is as follows:

(21) $$\begin{array}{l} \Delta E\min=\mathrm{Max}\left|e\cos(\eta_{i}-\beta )+\sqrt{{(r_{p}-((\frac{2}{n}\sum_{i=1}^{n}r_{i}\cos\eta_{i})\cos\eta_{i}+(\frac{2}{n}\sum_{i=1}^{n}r_{i}\sin\eta )\sin\eta_{i}))}^{2}-{(\mathrm{esin}(\eta_{i}-\beta ))}^{2}}\right|\\ -\mathrm{Min}\left|e\cos(\eta_{i}-\beta )+\sqrt{{(r_{p}-((\frac{2}{n}\sum_{i=1}^{n}r_{i}\cos\eta_{i})\cos\eta_{i}+(\frac{2}{n}\sum_{i=1}^{n}r_{i}\sin\eta_{i})\sin\eta_{i}))}^{2}-{(\mathrm{esin}(\eta_{i}-\beta ))}^{2}}\right| \end{array}$$

### 5.2. The Specific Process of the Algorithm and Its Implementation

Since the center of the least squares model involves a nonlinear solution, the solution is more complicated. The current mainstream linearization method is to evenly distribute the sampling points and take the number as an even number. The obtained center O (*P*_x0_, *P*_y0_) is as follows:

(22) $$p_{x0}=\frac{1}{n}\overset{n}{\underset{i=1}{\sum }}X_{i}\;\;p_{y0}=\frac{1}{n}\overset{n}{\underset{i=1}{\sum }}Y_{i}$$

The above center model is only suitable for the roundness evaluation of the whole circle. The center deviation is large when the sampling points are not round and the distribution is not uniform. Based on this, three points A (*P*_x1_, *P*_y1_), B (*P*_x2_, *P*_y2_), and C (*P*_x3_, *P*_y3_) with roughly uniform distribution in the effective sampling points are selected for initial fitting, and the center O (*P*_x0_, *P*_y0_) of the circle is used as the reference center as shown below:

(23) $$P_{x0}=\frac{\left(P_{y1}-P_{y2}\right)\left(P_{y1}P_{y2}+P_{x3}{}^{2}\right)+\left(P_{y2}-P_{y2}\right)\left(P_{y2}P_{y3}+P_{x1}{}^{2}\right)-\left(P_{y1}-P_{y3}\right)\left(P_{y3}P_{y1}+P_{x2}{}^{2}\right)}{2P_{y1}\left(P_{x3}-P_{x2}\right)+2P_{y2}\left(P_{x1}-P_{x3}\right)+2P_{y3}\left(P_{x2}-P_{x1}\right)}\;P_{y0}=\frac{\left(P_{x1}-P_{x2}\right)\left(P_{x1}P_{x2}+P_{y3}{}^{2}\right)+\left(P_{x2}-P_{x3}\right)\left(P_{x3}P_{x2}+P_{y1}{}^{2}\right)-\left(P_{x1}-P_{x3}\right)\left(P_{x1}P_{x3}+P_{y2}{}^{2}\right)}{2P_{x1}\left(P_{y3}-P_{y2}\right)+2P_{x2}\left(P_{y1}-P_{y3}\right)+2P_{x3}\left(P_{y2}-P_{y1}\right)}$$

**Figure 7:**
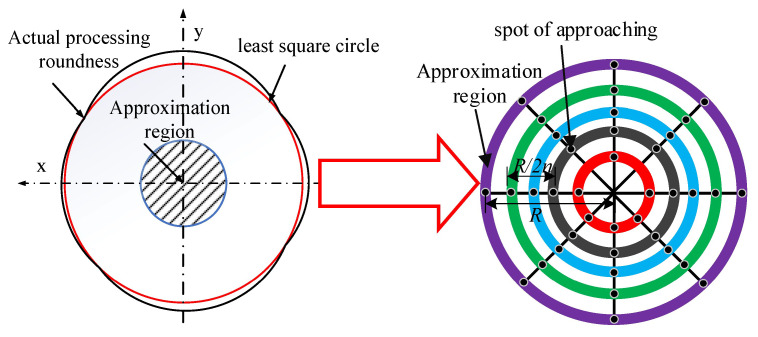
Schematic diagram of sphericity approximation method.

The center of the circle determined by the above formula is set as the search center of the algorithm, and an initial limited area of the circular section of the ceramic ball with a radius of *R* is created. The limited area is refined. Specifically, *n* lines passing through the center of the circle and equally dividing the initial area are first made and then *n* concentric circles are made with *R* − *R/n* as the radius. The intersection point of the two is the algorithm approximation point. The corresponding approximation region is shown in Figure 7.

The size of *R* is judged by the least square method. In this paper, the value of the least square roundness error is used as the specific value of *R*. The circular approximation region is minimized and divided many times. The polar angle of the divided minimum region is *τ*_i_, and the polar diameter is *R*/*n*. The coordinate *O*_x_ (*x*_i_, *y*_i_) of the height refinement point can be expressed as the following mathematical model:

(24) $$x_{i}=(x_{0}+R/n)cos\left(n\tau_{i}\right)\;y_{i}=(y_{0}+R/n)Rsin\left(n\tau_{i}\right)$$

Assuming that the measured points on the surface of the measured sphere are *P*_i_ (*X*_i_, *Y*_i_), the distribution and number of measured points are not limited. Taking the center of the reference circle as the search center, the appropriate search area size and search step are determined. Taking the points to be searched in these areas as the center of the circle, the maximum radius *R*_max_, the minimum radius *R*_min,_ and the radius range Δ*R* in each area are calculated.

According to the values of the maximum radius *R*_max_, the minimum radius *R*_min,_ and the radius range Δ*R* obtained by the above calculation, the above values calculated in the *n*^2^ regions of the sphere refinement are compared by different evaluation criteria. The following results can be obtained:

(1) The minimum value of the maximum radius value *R*_max_ in each region is the minimum circumscribed circle radius of the ceramic ball, which is set to *R*_Cmax_. At this time, the search point in the region is the center of the minimum circumscribed circle, and the corresponding minimum radius value is set to *R*_Cmin_. According to the evaluation standard of the minimum circumscribed circle method, the error value *E*_C1_ at this time is known;
(2) The maximum value of the minimum radius value *R*_min_ in each region is the maximum inscribed circle radius of the ceramic ball, which is set to *R*_imin_. At this time, the search point in the region is the maximum inscribed circle center, and the corresponding maximum radius is set to *R*_imax_. According to the evaluation standard of the maximum inscribed circle method, the error value *E*_C2_ is known at this time;
(3) The range Δ*r* of the radius value in the approximation area is calculated and compared, and the minimum value is the minimum value of the contour of the measured circle. The error value *E*_C3_ can be known from the corresponding minimum zone evaluation method standard.

The calculation formulas of the above roundness error calculation results in *E*_C1_, *E*_C2,_ and *E*_C3_ are as follows:

(25) $$\left\{ \begin{array}{l} E_{C1}=R_{Cmax}-R_{Cmin}\\ E_{C2}=R_{imax}-R_{imin}\\ E_{C3}=\min\{\Delta R\} \end{array}\right.$$

Comparing the values of *E*_C1_, *E*_C2_, and *E*_C3_, the minimum value is selected as the roundness error calculation result of this algorithm.

To avoid the low accuracy of the calculated results and the possibility of locally optimal solutions, the constraint accuracy *α* is considered, which is defined as the deviation between the minimum value of the radius range of each search point in the approximation area and its adjacent value. The selection of this value should be based on the value of the least squares roundness error as the standard R, which usually satisfies the relationship of *R*/*n*^2^.

**Figure 8:**
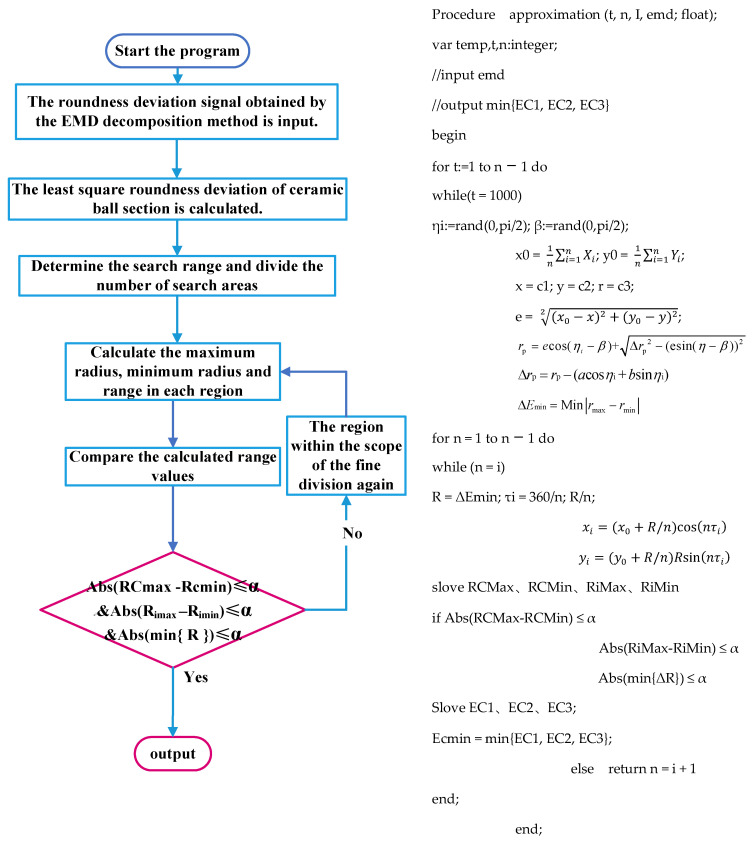
Algorithm flow chart and related pseudo-codes.

The above calculation is realized by MATLAB, and the specific implementation process and pseudo-code are as follows (Figure 8):

## 6. Experimental Verification and Algorithm Accuracy Analysis

### 6.1. Experimental Design

**Figure 9:**
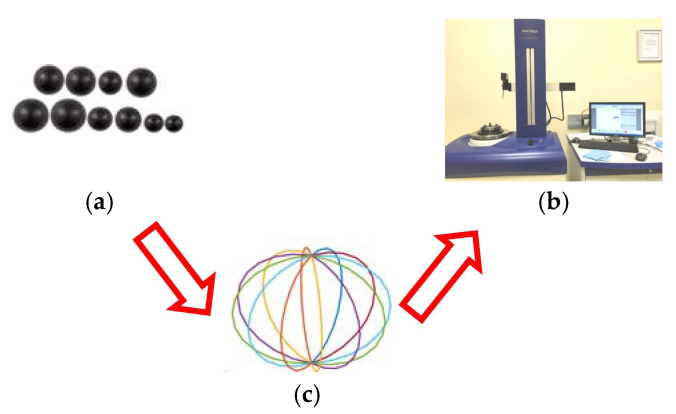
Sample measurement flow chart. (**a**) silicon nitride ball; (**b**) Taylor roundness instrument; (**c**) The selected section schematic diagram.

Based on the spherical approximation algorithm of ceramic balls mentioned above, to verify its accuracy, this paper uses silicon nitride ceramic balls as the main research object. The number of samples participating in the experiment is divided into 10 groups. Each group of samples is processed under the condition of sufficient change of rotation angle. The sample is repeated for 6 times of the diameter measurement to take the average value, and the corresponding specification parameters are shown in Table 1 and Table 2 [47]. Taking 12 cross-sections for each sample, the Taylor Hobson roundness instrument is used to measure the roundness and uniformly sample the outer surface distribution of the contour of each cross-section. The filtering method used for roundness measurement is Gaussian filtering. The cut-off value of the filter is 1~15 upr, while the equal angle sampling does not use the filter. The measuring probe type is *Φ* 2 mm. The specific sampling flow chart is shown in Figure 9.

### 6.2. Uncertainty Analysis

Based on the above experiments, in the actual measurement process, due to the influence of external ambient temperature, instrument measurement accuracy, offset, and tilt in the sampling process on the measurement results, it is necessary to analyze the uncertainty of this experiment. In order to ensure the accuracy and reliability of the analysis, this paper uses the GUM method to evaluate the uncertainty.

Taking the original signal measurement value shown in Figure 6 as the research object, the uncertainty of each sampling point is calculated, and the main factors affecting the synthetic uncertainty *u*_0_ of each sampling point of the original signal are analyzed. The specific process steps are as follows:

(1) Considering the operation error in the measurement process, the coordinate measurement of each sampling point is repeated 10 times, that is, m = 10, and the corresponding uncertainty *u*_1_ is obtained as follows;
(2) According to the relevant technical documents, the maximum allowable deviation of the instrument is 0.02 μm, that is, the standard deviation σ is 0.02 μm, so that the measured value of the sampling point obeys the normal distribution, and the corresponding confidence factor K is 1.96. The uncertainty *u*_2_ caused by the error of the instrument‘s own indication is usually related to the standard deviation *σ,* and the confidence factor *K* as follows:

(26) $$u_{2}=\frac{\sigma }{K}$$

(3) In the process of sampling the outer surface of the sphere by the instrument, the uncertainty is caused by temperature *u*_3_ = 0 because the external environment temperature is basically kept constant at 20 °C;
(4) In the actual sampling process, the probe basically does not produce offset and tilt, so the uncertainty caused by this item is *u*_4_ = 0;
(5) The above uncertainty is combined, and the corresponding combined single-point uncertainty formula is as follows:

(27) $$u=\sqrt{u_{1}{}^{2}+u_{2}{}^{2}+u_{3}{}^{2}+u_{4}{}^{2}}$$

(6) For the center of the ceramic sphere, the uncertainty generated in the detection process can usually be expressed by the following model:

(28) $$\left\{ \begin{array}{l} u_{x_{a}}=\frac{x_{a}}{\sqrt{m}}\\ u_{y_{b}}=\frac{y_{b}}{\sqrt{m}} \end{array}\right.$$

(7) Due to the large number of sampling points set in this paper, the difference εab between *u*_xa_ and *u*_ya_ of the center of the circle will be infinitely reduced to approximately zero in the actual measurement process, and the combined standard uncertainty *u*_c_ formed on this basis is as follows:

(29) $$\begin{array}{l} {u_{c}}^{2}={\left(\frac{\partial \Delta E_{min}}{\partial x_{a}}u_{xa}\right)}^{2}+{\left(\frac{\partial \Delta E_{min}}{\partial y_{b}}u_{\mathrm{yb}}\right)}^{2}+2\frac{\partial \Delta E_{min}}{\partial x_{a}}\frac{\partial \Delta E_{min}}{\partial y_{b}}\varepsilon_{ab}u_{xa}{}_{\mathrm{yb}}+{\left(\frac{\partial \Delta E_{min}}{\partial \eta_{max}}u_{\eta_{max}}\right)}^{2}\\ +{\left(\frac{\partial \Delta E_{min}}{\partial r_{max}}u_{r_{max}}\right)}^{2}+{\left(\frac{\partial \Delta E_{min}}{\partial \eta_{min}}u_{\eta_{min}}\right)}^{2}+{\left(\frac{\partial \Delta E_{min}}{\partial r_{min}}u_{r_{min}}\right)}^{2} \end{array}$$

### 6.3. Analysis of Experimental Results

The roundness deviation *E*c of the 12 cross-section profiles corresponding to each of the above 10 samples was detected by the Taylor roundness tester. Each cross-section was repeatedly sampled 10 times, and the mean value was taken as the roundness error value *E*c under the cross-section. On this basis, the differences between the roundness of each cross-section were compared, to verify whether the roundness of the different cross-sections of the finished ball processed by the ceramic ball under the condition of a sufficient change of the rotation angle during the lapping process was close to the same.

**Figure 10:**
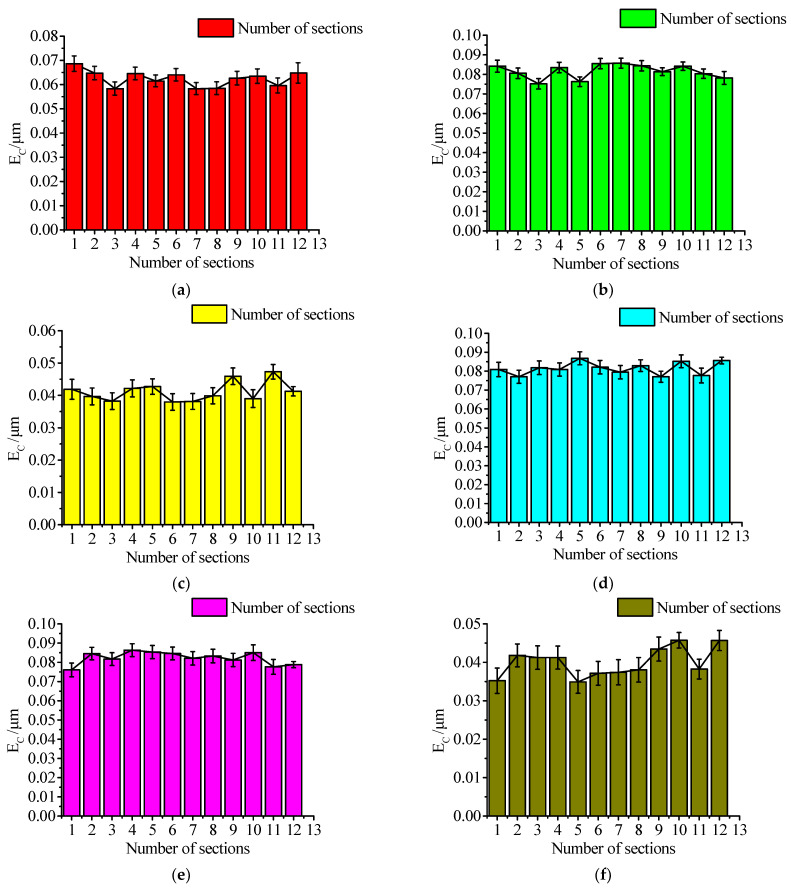

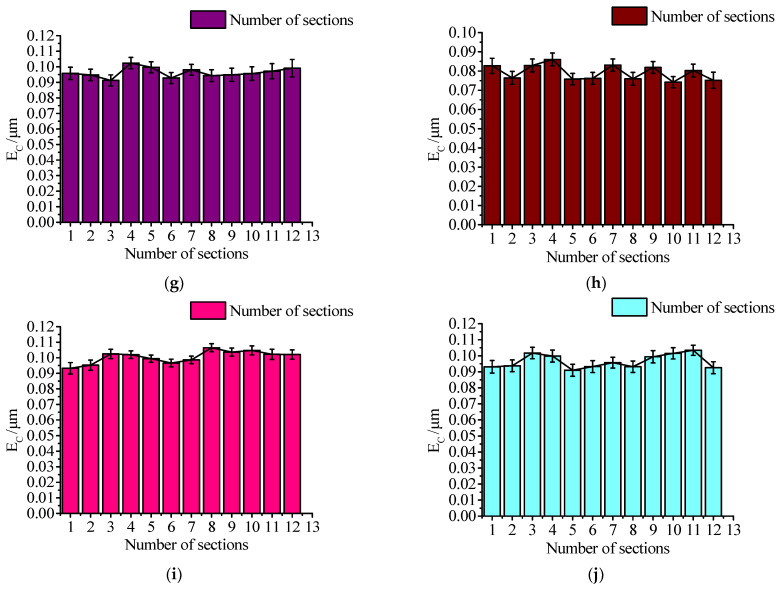
Comparison of roundness deviation of ceramic balls under different samples and different sections of the same sample. (**a**) The E_C_ values under different cross sections of Sample 1; (**b**) The E_C_ values under different cross sections of Sample; (**c**) The E_C_ values under different cross sections of Sample 3; (**d**) The E_C_ values under different cross sections of Sample 4; (**e**) The E_C_ values under different cross sections of Sample 5; (**f**) The E_C_ values under different cross sections of Sample 6; (**g**) The E_C_ values under different cross sections of Sample 7; (**h**) The E_C_ values under different cross sections of Sample 8; (**i**) The E_C_ values under different cross sections of Sample 9; (**j**) The E_C_ values under different cross sections of Sample 10.

Each experimental sample fully considers the impact of uncertainty in the measurement process, and the results are shown in Figure 10.

From the experimental results shown in Figure 10 above, it can be seen that the roundness deviation of different cross-sections selected by the same ceramic ball sample is basically close, and the corresponding standard deviation SD value is basically stable at approximately 0.03 μm, that is, the roundness error value fluctuates less, and the data concentration degree is higher. This shows that the accuracy of different cross-section shapes selected by the same ceramic ball sample tends to be consistent, which undoubtedly proves the results of the previous simulation part; the larger the rotation angle variation of the ceramic ball during the lapping process, the more sufficient the spin motion during the lapping process and the more consistent processing effect can be achieved on each surface of the ball, so as to ensure the reliability of the contour shape signal detected under different sections.

**Figure 11:**
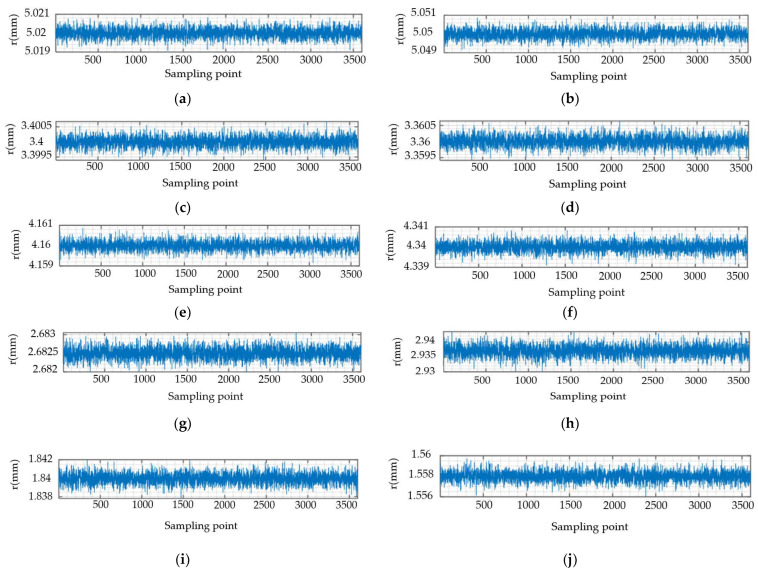
Original signal of ceramic ball surface. (**a**) Sample 1 original signal diagram; (**b**) Sample 2 original signal diagram; (**c**) Sample 3 original signal diagram; (**d**) Sample 4 original signal diagram; (**e**) Sample 5 original signal diagram; (**f**) Sample 6 original signal diagram; (**g**) Sample 7 original signal diagram; (**h**) Sample 8 original signal diagram; (**i**) Sample 9 original signal diagram; (**j**) Sample 10 original signal diagram.

Based on the above conclusions, 10 samples are detected respectively, and the mean values of 12 cross-section contour points detected by each sample are calculated. The calculated average signal is used as the original signal of the outer contour of the ceramic ball, as shown in Figure 11 below.

**Figure 12:**
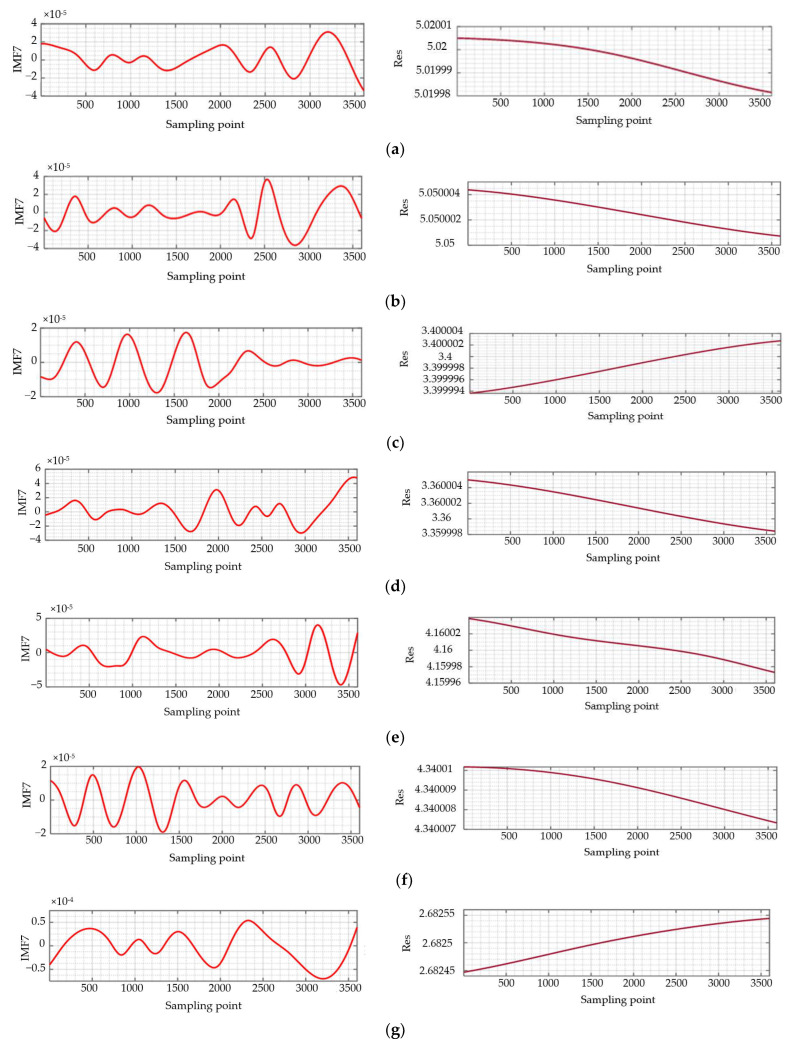

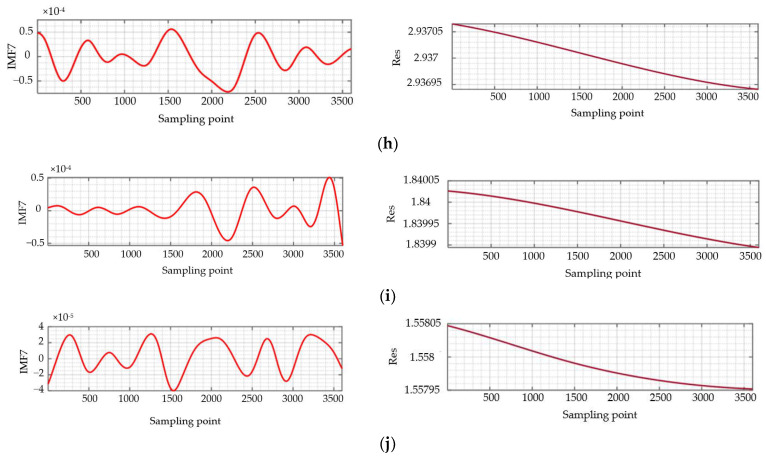
EMD decomposition results of ceramic ball surface original signal. (**a**) Decomposition results of Sample 1; (**b**) Decomposition results of Sample 2; (**c**) Decomposition results of Sample 3; (**d**) Decomposition results of Sample 4; (**e**) Decomposition results of Sample 5; (**f**) Decomposition results of Sample 6; (**g**) Decomposition results of Sample 7; (**h**) Decomposition results of Sample 8; (**i**) Decomposition results of Sample 9; (**j**) Decomposition results of Sample 10.

The original signal of each sample detected in Figure 11 is processed by the empirical mode decomposition method EMD, mentioned in this paper. For the initial decomposition of the intrinsic mode function IMF, it can be seen from the previous text that the number of intersections n with the zero-frequency component Z_j_(t) basically satisfies the double relationship with the number of waveforms *K*c. Therefore, after obtaining the number of intersection points n, the number of waveforms *K*c of each Intrinsic Mode Function can be deduced. According to the relevant instructions of the EMD method, the number of waveforms *K*c = 15 is used as the main criterion. The signal with the number of waveforms *K*c greater than this value in the Intrinsic Mode Functions IMF is set as the precision error interference signal, and it is eliminated. The IMF7 and residual error Res obtained by the final decomposition are shown in Figure 12.

After the original signal decomposition of the above samples is completed, the remaining intrinsic mode function IMF7 and the residual error Res are reconstructed to obtain the roundness error signal of the ceramic ball, and the signal is put into the roundness approximation algorithm as the initial surface detection signal. It can be seen from the previous text that the algorithm takes the least squares center corresponding to the cross-section of the ceramic ball as the reference point, and sets the radius *R* of the approximation region to be equal to the circle of the least squares roundness error Δ*E*_min_. Starting from the edge of the region, it is divided into 120 fine points of different sizes, and the fine points inside the minimum approximation region formed by each division are the center of the circle. The maximum value *R*_max_ and the minimum value *R*_min_ of the distance corresponding to the roundness error signal are calculated, and the corresponding range Δ*R* is obtained.

From the previous introduction of the algorithm flow, it can be seen that the calculated maximum *R*_max_, minimum *R*_min,_ and range Δ*R* are solved by the minimum circumscribed circle evaluation method, the maximum inscribed circle evaluation method, and the minimum area evaluation method. The corresponding roundness errors *E*_C1_, *E*_C2,_ and *E*_C3_ are obtained by setting the constraint accuracy α of the algorithm to *R*/120^2^.

**Figure 13:**
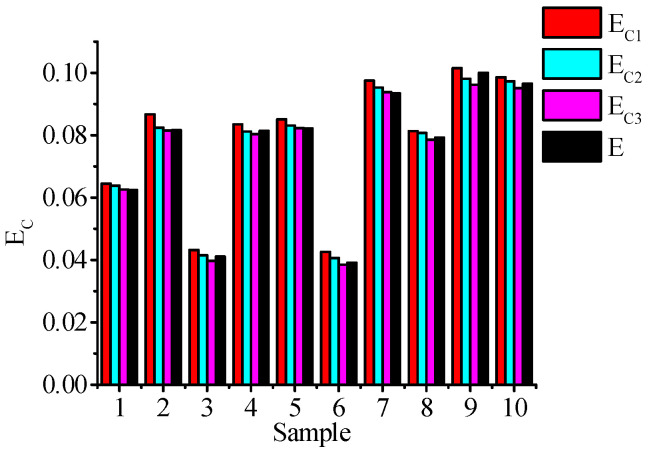
Comparison of calculation results and roundness instrument test results.

The calculated roundness error values *E*_C1_, *E*_C2_, and *E*_C3_ are shown in Table 3. Comparing it with the roundness instrument test result E, the result shown in Figure 13 is obtained.

By analyzing Table 3 and Figure 13, it can be seen that the roundness error values *E*_C1_, *E*_C2,_ and *E*_C3_ calculated by samples 1 to 10 are different, which indicates that there are some differences in the results obtained by the calculation method under different evaluation criteria. The error *E*_C1_ obtained by the minimum circumcircle evaluation standard is the largest, and the error *E*_C3_ obtained by the minimum zone evaluation standard is the smallest. The relationship between the three is *E*_C1_ > *E*_C2_ > *E*_C3_, and the value of *E*_C3_ is the closest to the result obtained by the roundness tester. The relative error between the two is maintained at (0.32~1.45%). Therefore, in the actual measurement process, the error *E*_C3_ obtained by the minimum zone evaluation standard should be used as the roundness error value obtained by this algorithm. At the same time, the error *E*_C3_ is basically consistent with the instrument-measured value *E*, indicating that the algorithm can better realize the accurate evaluation of the form deviation of the outer surface of the ceramic ball.

Similarly, the original signal collected on the surface of the ceramic ball is used as the input signal to be solved by the mainstream algorithms, such as the least circumscribed circle method, the least square method, the maximum inscribed circle method, and the minimum region method. The roundness error calculated by the minimum circumscribed circle method is *E*_a1_, the roundness error calculated by the least square method is *E*_a2_, the roundness error calculated by the maximum inscribed circle method is *E*_a3_, and the roundness error calculated by the minimum region method is *E*_a4_.

**Figure 14:**
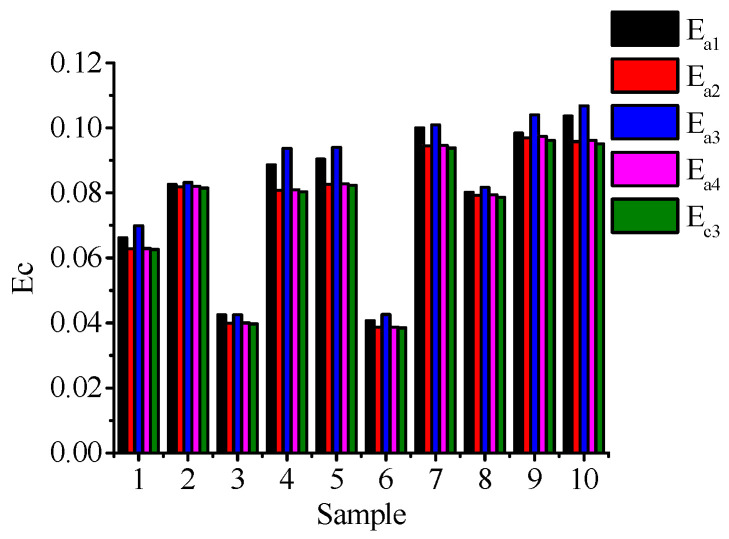
Comparison of calculation results of different methods.

The calculated results are compared with the roundness error value *E*_C3,_ obtained by the method described in Table 4, and the results are shown in Figure 14.

Combined with Table 4 and Figure 14, it can be clearly seen that the roundness error obtained by the method described in this paper is quite different from the roundness error obtained by the traditional method. The error *E*_a3_ obtained by the maximum inscribed circle method is the largest, followed by the error Ea1 obtained by the minimum circumscribed circle method. The error Ea obtained by the least square method is the minimum value calculated by the traditional method, but the value is still less than the roundness error value *E*_C3_ obtained by the method described in this paper. The relative error between the two ranges from 1.39% to 10.1%. At the same time, compared with Table 3 and Table 4, it can be seen that for the same evaluation method, the calculation method in this paper can reduce the corresponding roundness error; among them, the mean error reduced by the minimum circumscribed circle method is 0.0087 μm, the mean error reduced by the maximum inscribed circle method is 0.0553 μm, and the mean error reduced by the minimum zone method is 0.00633 μm. Therefore, the calculation accuracy of the method described in this paper is higher than that of several existing, traditional algorithms. At the same time, it shows that the algorithm can better realize the accurate evaluation of the form deviation of the outer surface of the ceramic ball, and it provides a great guiding value in the evaluation of the shape accuracy of the ceramic ball processed under the condition of a sufficient change of the rotation angle.

## 7. Conclusions

This paper proposes an evaluation algorithm for the shape error of the ceramic sphere’s outer surface applied to the lapping trajectory’s uniform envelope. The main factors to improve the uniformity of the lapping trajectory on the surface of the ceramic sphere are verified by simulation analysis and experiment, that is, the variation range of the rotation angle of the sphere. When the value of ∆ *δ* is larger, the trajectory of the surface of the sphere is more uniform, and the envelope of the sphere can be realized. The different sections of the sphere can achieve more consistent processing, that is, the roundness deviation error of different sections is small. The experimental results show that the corresponding standard deviation SD value is basically stable at approximately 0.03 μm. Combining the empirical mode decomposition method with the roundness approximation algorithm, the roundness error signal is extracted by the empirical mode decomposition method and used as the input signal of the roundness approximation algorithm. The experimental results show that the method has a great advantage in accuracy compared with the traditional algorithm and is basically consistent with the results obtained by the instrument detection.

In summary, the method designed in this paper fills the gap in the field of ceramic balls with uniform distribution of lapping trajectories to a certain extent and provides a new idea for the accurate evaluation of the spherical error of ceramic balls.

## Figures and Tables

**Table 1:** Performance parameters of silicon nitride ceramic ball.

Mechanical Property	Parameter Value
Density/(g·cm^–3^)	3.2 × 10^3^
Elastic Modulus/Gpa	310
Hardness/HRC	94
Poisson’s ratio	0.26
Fracture toughness/(MPa·m^–2^)	7.0
Compressive strength/Pa	420
Thermal expansion coefficient/(10^–6^·K^–1^)	3.0 × 10^6^

**Table 2:** Experimental sample specifications.

Serial Number	Diameter	Accuracy Grade
1	10.043	G3
2	10.111	G3
3	6.832	G3
4	6.721	G3
5	8.327	G3
6	8.683	G3
7	5.365	G5
8	5.874	G5
9	3.691	G5
10	3.116	G5

**Table 3:** Roundness error calculation results.

Sample Number	*E*_C1_/μm	*E*_C2_/μm	*E*_C3_/μm	*E*_C_/μm
1	0.0645	0.0638	0.0626	0.0624
2	0.0867	0.0824	0.0815	0.0816
3	0.0432	0.0415	0.0397	0.0411
4	0.0835	0.0812	0.0804	0.0814
5	0.0851	0.0831	0.0823	0.0822
6	0.0426	0.0406	0.0385	0.0391
7	0.0975	0.0953	0.0938	0.0934
8	0.0813	0.0807	0.0786	0.0792
9	0.1015	0.0981	0.0962	0.1000
10	0.0986	0.0973	0.0951	0.0965

**Table 4:** Roundness error calculation results of different methods.

Sample Number	*E*_a1_/μm	*E*_a2_/μm	*E*_a3_/μm	*E*_a4_/μm	*E*_c_/μm
1	0.0662	0.0628	0.0699	0.0629	0.0626
2	0.0826	0.0819	0.0832	0.0820	0.0815
3	0.0425	0.0399	0.0425	0.0400	0.0397
4	0.0886	0.0808	0.0937	0.0810	0.0804
5	0.0904	0.0826	0.0940	0.0828	0.0823
6	0.0407	0.0387	0.0426	0.0387	0.0385
7	0.1000	0.0945	0.1009	0.0946	0.0938
8	0.0802	0.0793	0.0817	0.0794	0.0786
9	0.0984	0.0970	0.1040	0.0974	0.0962
10	0.1036	0.0958	0.1068	0.0962	0.0951

## Data Availability

The data are available from the authors.

## Abbreviations

[*O*_p_ *X*_d_ *Y*_d_ *Z*_d_]	Fixed coordinate system
[*O*_p_ *X*_z_ *Y*_z_ *Z*_z_]	Rotary coordinate system
[*O*_z_, *X*_s_ *Y*_s_ *Z*_s_]	Rotation coordinate system of ceramic ball
*δ*	Rotation angle of ceramic ball
[*O*_z,_ *X*_s,_ *Y*_s_, *Z*_s_]	The coordinate system in the state of spin angle change
*P*_N_	Initial matrix
tran(*u*, *δ*)	Coordinate change matrix of ceramic ball
*ω*_1_, *ω*_2_, *ω*_3_	Angular velocity of contact point
*ω_z_*	Rotational angular velocity of ceramic balls
*ω*_b_	Projection of angular velocity on x axis
*ω*_c_	Projection of angular velocity on y axis
*R*_B_, *R*_C_	The distance from the contact point to the radius of rotation of the sphere
*r*	The radius value of ceramic ball
*η*	Groove angle
Δ*F*	Rotation amplitude matrix
Δ*t*	The unit sampling time of the trajectory points on the sphere surface
*C*	Real-time state matrix of sphere rotation angle in grinding process
*P*_N_(i + 1)	The trajectory coordinates of the ceramic ball obtained after turning any angle during the grinding process
*δ*(*i*)/Δ*δ*	The variation range of rotation angle in unit time
*m*_1_	Upper envelope line
*m*_2_	Lower envelope line
*D*	The difference between the time series signal and the mean value
L	Stopping function
*P*_j_ (t)	Data set from initial high-frequency IMF to minimum low-frequency IMF
*P*_j_ (t)	IMF classification
*Q*_m_ (t)	Residual error
*Z*_j_(t)	Zero-frequency component
IMF	Intrinsic Mode Functions
*K*_c_	Number of waveforms
*O*_L_	The center of the least squares circle
*C*_i_	The actual measured contour points
Δ*E*_min_	The roundness deviation of the sphere obtained by the least square method
*e*	The distance from the center of Rotation O to the circle OL
*r_p_*	The distance from any point on the actual contour line to the center OL
*β*	The initial angle between rp and polar axis
Δ*r*_p_	The distance deviation value in the model
*R*	Approximation region radius of the algorithm
*O*_x_	Highly fine points
*R*_max_	The maximum radius in each minimum partition region
*R*_min_	The minimum radius in each minimum partition region
Δ*R*	The radius range in each minimum partition area
MZM	Minimum zone method
LSM	Least square method
MCM	Minimum circumcircle method
MIC	Maximum inscribed circle method
*E*_C1_	The algorithm is based on the MCM to evaluate the roundness deviation obtained by the standard
*E*_C2_	The algorithm is based on the MIM to evaluate the roundness deviation obtained by the standard
*E*_C3_	The algorithm is based on the MIC to evaluate the roundness deviation obtained by the standard
*E*_c_	Roundness deviation value detected by instrument
*E*_a1_	The roundness deviation calculated by the MCM
*E*_a2_	The roundness deviation calculated by the LSM
*E*_a3_	The roundness deviation calculated by the MIC method
*E*_a4_	The roundness deviation calculated by the MZM

## References

[1] Clarke I., Pezzotti G., Lakshminarayanan A., Burgett M.M., Donaldson T. (2016). Silicon Nitride Bearings: An Alternative to Oxide Ceramics in Total Hip Arthroplasty. Orthop. Proc..

[2] Tian J., Wu Y., Sun J., Xia Z., Ren K., Wang H., Li S., Yao J. (2022). Thermal Dynamic Exploration of Full-Ceramic Ball Bearings under the Self-Lubrication Condition. Lubricants.

[3] Zhang X., Wu D., Xia Z., Li Y., Wang J., Han E.-H. (2022). Characteristics and mechanism of surface damage of hybrid ceramic ball bearings for high-precision machine tool. Eng. Fail. Anal..

[4] Sun J., Chen W., Yao J.M., Li S.H., Tian J.X. (2023). Analysis of the Lapping trajectory of Si3N4 Ceramic Balls and Research on Its Effect Mechanism of Surface Quality. Surf. Technol..

[5] Zhou F.F., Yuan J.L., Yao E.F., Lv B.H., Ruan D.N. (2019). Review on Ultra-percision Machining Technology of Precision Balls. Chin. J. Mech. Eng..

[6] Xiao X.L., Yan Q.S., Lin H.T., Jiao J.H., Liu J. (2018). Research Progress of Silicon Nitride Ceramic Ball Lapping and Polishing Technology. J. Guangdong Univ. Technol..

[7] Zmarzły P. (2020). Multi-Dimensional Mathematical Wear Models of Vibration Generated by Rolling Ball Bearings Made of AISI 52100 Bearing Steel. Materials.

[8] Zmarzły P. (2022). Analysis of Technological Heredity in the Production of Rolling Bearing Rings Made of AISI 52100 Steel Based on Waviness Measurements. Materials.

[9] Yuan C., Wu C., Fang X., Liao N., Tong J., Yu C. (2022). Effect of Slurry Concentration on the Ceramic Ball Grinding Characteristics of Magnetite. Minerals.

[10] Dong M.G., Yong F.L. (2019). Overview of extreme manufacturing. Int. J. Extrem. Manuf..

[11] Wang D.X., Wen X.L., Qiao G.F. (2018). Estimation of uncertainty in measuring the workpiece circularity error. Opt. Precis. Eng..

[12] Cai Z., Wang J.L., Lv M.F., Zhu L.B. (2020). Roundness deviation Assessment Based on Improved Cuckoo Search Algorithm. Modul. Mach. Tool Autom. Manuf. Tech..

[13] Zhang K., Wang D.W., Li S.H., Sun J., Wu Y.H. (2019). Materail removal mode of lapping Si3N4 balls. Diam. Abras. Eng..

[14] Wu Y.H., Sha Y., Li S.H., Tian J.X. (2021). Wear mode transition in micro-abrasive of silicon nitride ceramic balls. Ordnance Mater. Sci. Eng..

[15] Zhou Z.Z., Yuan J.L., Lv B.H., Zheng J.J. (2009). Roundness deviation Correction for Ceramic Ball Lapping Process. Bearing.

[16] Zhang B., Uematsu T., Nakajima A. (1998). High Efficiency and Precision Grinding of Si_3_N_4_ Ceramic Balls Aided by Magnetic Fluid Support Using Diamond Wheels. JSME Int. J. Ser. C Mech. Syst. Mach. Elem. Manuf..

[17] Zhang B., Nakajima A. (2000). Spherical surface generation mechanism in the lapping of balls for ultraprecision ball bearing. Proc. Inst. Mech. Eng..

[18] Lee R.-T., Hwang Y.-C., Chiou Y.-C. (2006). Lapping of ultra-precision ball surfaces part I: Concentric V-groove lapping system. Int. J. Mach. Tools Manuf..

[19] Lee R.T., Yih C.H., Chiou Y.C. (2006). Lapping of ultra-precision ball surfaces. Part II. Eccentric V-groove lapping system. Int. J. Mach. Tools Manuf..

[20] Mei J., Huang Q.X., Chen J.G., Cheng R.G., Zhang L.S., Fang C.Z., Wang C.Q., Cheng Z.Y. (2020). A simple asymptotic search method for estimation of minimum zone sphericity error. AIP Adv..

[21] Xuyi S., Ming L. (2019). A Sphericity Error Assessment Application Based on Whale Optimization Algorithm. IOP Conf. Ser. Mater. Sci. Eng..

[22] Gogolewski D., Bartkowiak T., Kozior T., Zmarzły P. (2021). Multiscale Analysis of Surface Texture Quality of Models Manufactured by Laser Powder-Bed Fusion Technology and Machining from 316L Steel. Materials.

[23] Gogolewski D., Zmarzły P., Kozior T., Mathia T.G. (2023). Possibilities of a Hybrid Method for a Time-Scale-Frequency Analysis in the Aspect of Identifying Surface Topography Irregularities. Materials.

[24] Ito S., Tsutsumi D., Kamiya K., Matsumoto K., Kawasegi N. (2020). Measurement of form error of a probe tip ball for coordinate measuring machine (CMM) using a rotating reference sphere. Precis. Eng..

[25] Jiang B.C., Xin D., Shilei B., Lulu W. (2022). Roundness error evaluation in image domain based on an improved bee colony algorithm. Mech. Sci..

[26] Dong Z.P., Huang F.G. (2011). Review of measurement and evaluation methods for Roundness Error. Tool Eng..

[27] Lei X.Q., Chang W.H., Xue Y.J., Li Y., Li J.X. (2008). Grid search algorithm for roundness deviation. Chin. J. Sci. Instrum..

[28] Yue L.L., Huang Q.X., Mei J., Cheng R.J., Zhang L.S., Chen L.J. (2020). Method for Roundness deviation Evaluation Based on Minimun Zone Method. J. Mech. Eng..

[29] Li F., Zhou F.F., Li X.L., Zhao P., Yuan J.L. (2015). Review on Roundness Error Separation Technique for Precision Bearings Balls. Bearing.

[30] Fenfen Z., Weifeng Y., Julong Y., Binghai L., Tianchen Z., Ping Z. (2021). Experimental study on lapping ceramic balls with variable-radius groove plate. Adv. Mech. Eng..

[31] Xiao X.-L., Li G.-X., Mei H.-J., Yan Q.-S., Lin H.-T., Zhang F.-L. (2020). Polishing of Silicon Nitride Ceramic Balls by Clustered Magnetorheological Finish. Micromachines.

[32] Zhao P., Guo W., Feng M., Lv B., Deng Q., Yuan J. (2013). A Novel Lapping Method for High Precision Balls Based on Variable-Radius V-Groove. J. Micro Nano-Manuf..

[33] Lei G., Xiaoke L., Yanchun Y., Yucong W., Xuzhe Y., Xinyu Z., Duanyang G., Yang L., Li L. (2022). A Modal Frequency Estimation Method of Non-Stationary Signal under Mass Time-Varying Condition Based on EMD Algorithm. Appl. Sci..

[34] Rekam M., Sekhar S.R., Raj J.A. (2022). Application of EMD based statistical parameters for the prediction of fault severity in a spur gear through vibration signals. Adv. Mater. Process. Technol..

[35] Zhongze L., Kang D., Huibin L., Guolin H., Canyi D., Zhuyun C. (2022). A Novel Impact Feature Extraction Method Based on EMD and Sparse Decomposition for Gear Local Fault Diagnosis. Machines.

[36] Meng D., Wang H., Yang S., Lv Z., Hu Z., Wang Z. (2022). Fault Analysis of Wind Power Rolling Bearing Based on EMD Feature Extraction. Comput. Model. Eng. Sci..

[37] Yongjian S., Shaohui L., Xiaohong W. (2021). Bearing fault diagnosis based on EMD and improved Chebyshev distance in SDP image. Measurement.

[38] Zhao X., Qin Y., Fu H., Jia L., Zhang X. (2021). Blind source extraction based on EMD and temporal correlation for rolling element bearing fault diagnosis. Smart Resilient Transp..

[39] Ma H., Li Z. (2021). Research on Bearing Life Prediction Method Based on EMD and Gray Model. IOP Conf. Ser. Mater. Sci. Eng..

[40] Shah A.K., Yadav A., Malik H. (2018). EMD and ANN based intelligent model for bearing fault diagnosis. J. Intell. Fuzzy Syst..

[41] (2004). Roundness and Waviness Deviation Measurement and Evaluation Method of Rolling Bearing Parts.

[42] Zhang G.W., Lv Q., Ma J.H., Liu Y. (2016). Roundness deviation Evaluation Based on Empirical Mode Decomposition. Tool Eng..

[43] Liu Z.T., Yang J.X., Zhao B. (2013). Study on Roundness Error Evaluation with Least-Squares Method Based on Nonlinear Optimization. Adv. Mater. Res..

[44] Lei X.Q., Li J.S., Duan M.D. (2010). Method for Roundness Error Evaluation Based on Geometry Optimization. J. Mech. Eng..

[45] Maja M., Jacek R., Joanna J. (2023). Tree position estimation from TLS data using hough transform and robust least-squares circle fitting. Remote Sens. Appl. Soc. Environ..

[46] Zheng P., Liu D., Zhao F., Zhang L. (2019). Statistical Evaluation Method for Cylindricity Deviation Using Local Least Squares Cylinder. Int. J. Precis. Eng. Manuf..

[47] Honghao L., Feng S., Tingxai D., Xuemin X. (2019). The Effect of Particle Size of Silicon Nitride Powder on Properties of Silicon Nitride Ceramic Balls. IOP Conf. Ser. Mater. Sci. Eng..

